# ATP-Dependent Ligases and AEP Primases Affect the Profile and Frequency of Mutations in Mycobacteria under Oxidative Stress

**DOI:** 10.3390/genes12040547

**Published:** 2021-04-09

**Authors:** Anna Brzostek, Filip Gąsior, Jakub Lach, Lidia Żukowska, Ewelina Lechowicz, Małgorzata Korycka-Machała, Dominik Strapagiel, Jarosław Dziadek

**Affiliations:** 1 Laboratory of Genetics and Physiology of Mycobacterium, Institute of Medical Biology of the Polish Academy of Sciences, 93-232 Lodz, Poland; abrzostek@cbm.pan.pl (A.B.); filip.gasior95@gmail.com (F.G.); limazuk5@gmail.com (L.Ż.); elechowicz93@gmail.com (E.L.); mkorycka@cbm.pan.pl (M.K.-M.); 2 BioMedChem Doctoral School of the University of Lodz and the Institutes of the Polish Academy of Sciences in Lodz, 90-237 Lodz, Poland; 3 Biobank Lab, Department of Molecular Biophysics, Faculty of Biology and Environmental Protection, University of Lodz, 90-237 Lodz, Poland; jakub.lach@biol.uni.lodz.pl (J.L.); dominik.strapagiel@biol.uni.lodz.pl (D.S.); 4 Institute of Microbiology, Biotechnology and Immunology, Faculty of Biology and Environmental Protection, University of Lodz, 90-237 Lodz, Poland

**Keywords:** *Mycobacterium*, ATP-dependent ligases, AEP primases, NHEJ, BER

## Abstract

The mycobacterial nonhomologous end-joining pathway (NHEJ) involved in double-strand break (DSB) repair consists of the multifunctional ATP-dependent ligase LigD and the DNA bridging protein Ku. The other ATP-dependent ligases LigC and AEP-primase PrimC are considered as backup in this process. The engagement of LigD, LigC, and PrimC in the base excision repair (BER) process in mycobacteria has also been postulated. Here, we evaluated the sensitivity of *Mycolicibacterium smegmatis* mutants defective in the synthesis of Ku, Ku-LigD, and LigC_1_-LigC_2_-PrimC, as well as mutants deprived of all these proteins to oxidative and nitrosative stresses, with the most prominent effect observed in mutants defective in the synthesis of Ku protein. Mutants defective in the synthesis of LigD or PrimC/LigC presented a lower frequency of spontaneous mutations than the wild-type strain or the strain defective in the synthesis of Ku protein. As identified by whole-genome sequencing, the most frequent substitutions in all investigated strains were T→G and A→C. Double substitutions, as well as insertions of T or CG, were exclusively identified in the strains carrying functional Ku and LigD proteins. On the other hand, the inactivation of Ku/LigD increased the efficiency of the deletion of G in the mutant strain.

## 1. Introduction

*Mycobacterium tuberculosis* (*Mtb*), the causative agent of tuberculosis (TB), is a leading bacterial pathogen claiming 1.5 million lives each year [1]. *Mtb* is an intracellular pathogen, and its life cycle includes long states of persistence. *Mtb,* as well as other pathogens, face a variety of harmful conditions during infection, caused by host defense mechanisms and drug treatments. As a very successful pathogen with outstanding adaptive properties, *Mtb* has developed a plethora of sophisticated mechanisms to subvert the host defense and to effectively enter and replicate in the harmful environment inside professional phagocytes, namely macrophages. Tubercle bacilli colonizing the mucosal surface of the lower respiratory epithelium are phagocytized by alveolar macrophages and exposed to various DNA-damaging assaults affecting the genome integrity, including reactive oxygen species (ROS) and reactive nitrogen intermediates (RNI). ROS and RNI are responsible for the oxidation or alkylation of bases, covalent linking of two bases, or elimination of a base leading to the creation of an abasic site. Prolonged exposure to toxic radicals may lead to single- or double-stranded breaks, affecting, if not repaired, DNA integrity and the replication process [2,3,4]. The nondistorting DNA lesions following the oxidation, deamination, or alkylation of bases in the DNA backbone caused by RNI/ROI are repaired preferentially by the base excision repair (BER) pathway. The single- and double-stranded breaks in mycobacteria are repaired by homologous recombination (HR), nonhomologous end joining (NHEJ), or single-stranded annealing (SSA) (for reviews, see [4,5,6]). The most faithful double-stranded break (DSB) repair pathway is HR, which requires the action of a second intact copy of a template. NHEJ can be mutagenic if the ends are modified by nucleases or polymerases before sealing. The SSA requires repeat flanking the DSB on both sides, and the sequence between the repeats is lost in the repair process.

Bacterial NHEJ was identified first in mycobacteria by in silico identification of the eukaryotic homologs of the end-bridging protein Ku and ATP-dependent DNA ligase [7,8]. Further, it was reported by in vitro experiments that the *Mtb* LigD protein exhibits ATP-dependent DNA ligase activity and is stimulated by its cognate Ku partner [9]. The construction of *Mycobacterium* (*Mycolicibacterium*) *smegmatis* mutants defective in the synthesis of the Ku and/or LigD protein allowed us to identify the role of NHEJ proteins in the recircularization of linear plasmid DNAs [10,11], as well as the recircularization of mycobacteriophage genomes, Corndog and Omega, during the transfection of mycobacteria [12]. It was also reported that NHEJ protects mycobacteria in the stationary phase against the harmful effects of ionizing radiation (IR) and desiccation [13]. 

Mycobacterial LigD consists of three autonomous enzymatic domains: polymerase (POL), phosphoesterase (PE), and ligase (LIG). LigD-POL is an AEP primase that can add both templated and nontemplated deoxynucleoside triphosphates (dNTPs) or ribonucleoside triphosphates (rNTPs) to DNA substrates [14,15]. The 3′ end-processing is supported by the LigD-PE domain with phosphodiesterase and monoesterase activities that can process 3′-phosphate-terminated DNA ends to 3′ OH [14,16]. LigD-LIG is an ATP-dependent DNA ligase stimulated by the presence of a single ribonucleotide [17]. 

The POL and PE domains are involved in DNA end processing before the ends are sealed by the LIG domain. Bacterial genomes often encode several putative Ku and LigD homologs. Four ATP-dependent DNA ligases (LigB, LigC_1_, LigC_2_, and LigD) are present in the saprophytic mycobacterial species *M. smegmatis* [18]. The main NHEJ ligase carrying the POL, LIG, and PE domains in *M. smegmatis* is LigD; however, an alternative NHEJ engaging Ku and LigC1 (single LIG domain protein) was also reported [18,19,20,21]. The genome of *M. smegmatis* also encodes four distinct primase polymerases classified as eukaryotic-like AEP primases, including the LigD-POL domain of LigD, which is not involved in the DNA replication process [22]. Except for LigD-POL, the function of AEP primases in mycobacteria has not yet been elucidated. MSMEG_6301 (Prim-PolC) is localized directly upstream of two DNA ligase genes (LigC1: *msmeg_6302* and LigC2: *msmeg_6304*). The ability of Prim-PolC and Msmeg_0597 to add templated and nontemplated nucleotides to primer templates and blunt ends, and their preference for rNTPs versus dNTPs were reported; however, the enzymes failed to process 5′-overhang DSBs in the NHEJ process [23]. The mycobacterial LigC-Prim-PolC complex was recently reported to fill short DNA gaps with ribonucleotides (rNTPs), followed by sealing of the resulting nicks [24]. It was shown that the LigC complex interacts with core BER enzymes in vivo to constitute an excision repair apparatus capable of repairing damaged bases and abasic sites. This observation, as well as the sensitivity of LigC/PrimC and LigD-deficient mutants to oxidative stress, revealed the dual role of ATP ligases and AEP primases in both excision and DSB repair in mycobacteria [24]. 

Here, by constructing and analyzing several *M. smegmatis* mutants defective in the synthesis of an individual or combinations of genes, ATP ligase(s) and AEP primases, we systematically evaluated the role of those enzymes in the accumulation of various mutations in mycobacterial genomes under the pressure of oxidative stress.

## 2. Materials and Methods

### 2.1. Bacterial Strains and Growth Conditions

Cultures of *Escherichia coli* were carried out at 37 °C for 18–20 h in liquid Luria–Bertani broth or agar plates supplemented with antibiotics: kanamycin (Bioshop, Burlington, MO, Canada) 50 μg/mL or ampicillin (Bioshop, Burlington, MO, Canada) 100 μg/mL. Cultures of *M. smegmatis* were carried out in 7H9 or 7H10 broth (Difco, Baltimore, MD, USA) with albumin dextrose growth supplement (AD) (Difco, Baltimore, MD, USA) and 0.05% Tween 80 (Bioshop, Burlington, MO, Canada) at 37 °C, and when necessary, the media were supplemented with antibiotics or other supplements or both at the following concentrations: kanamycin (Sigma Aldrich, St. Louis, MO, USA) 25 μg/mL, sucrose (Bioshop, Burlington, ON, Canada) 2%; CHP (Sigma Aldrich, St. Louis, MO, USA) 0.5 ng/mL. 

A list of *M. smegmatis* strains used in this study is presented in Table S1. The bacterial cells were cultured in 7H9/AD/Tween medium to the optical density (OD_600_) ≤ 1.0. Aliquots of these seed cultures were inoculated in fresh 7H9 broth supplemented with AD/Tween at a starting OD_600_ of 0.05. The cultures were incubated at 37 °C for 6 days, and [11] the samples of cultures were treated with DNA-damaging agents. To assess the number of the colony-forming units, samples were serially diluted in 1 × PBS (phosphate-buffered saline) with 0.05% Tween buffer, plated on 7H10/AD/Tween medium, and incubated at 37 °C for 2 to 3 days until visible colonies were obtained. Each experiment was performed at least in triplicate.

### 2.2. Gene Cloning Strategies

Standard molecular biology protocols were used for all cloning strategies [25]. The PCR product of the *sacB* gene with its natural promoter was obtained by using thermostable AccuPrime Pfx DNA polymerase (Invitrogen, Carlsbad, CA, USA) and primarily cloned into a blunt vector (pJET1.2; Thermo Fisher, Vilnius, Lithuania), sequenced, and then released by digestion with NotI/XbaI and ligated into the NotI/XbaI restriction sites of the final integrative vector pMV306Km, carrying an *attP* sequence and the integrase gene, allowing integration of the whole plasmid into the single *attB* site in the *M. smegmatis* chromosome. The integration was confirmed by PCR analysis. The mutants were used to estimate the mutation rate. All plasmids and oligonucleotides used in this work are listed in Supplementary Table S2.

### 2.3. Construction of Gene-Replacement Mutants

The construction of *M. smegmatis* mutants Δ*ku*, Δl*igD*, Δ*ku-ligD*, and Δ*ligC1-ligC2-primC* was described previously [11,24]. The mutant defective in the synthesis of all the investigated proteins (Δ*ku-ligD*-*ligC1-ligC2-primC*) was engineered by a replacement of the *ligC1-ligC2-primC* genes with mutated genes in the *M. smegmatis* Δ*ku-ligD* background. The genotype of the resultant mutant was verified by PCR amplification and Southern blot (Figure S1).

### 2.4. Phenotypic Analysis of M. Smegmatis Strains

*M. smegmatis* mc^2^ 155 wild type (ATCC 700084) and its mutants were grown to the late-stationary phase (5 days reaching optical density (OD_600_ nm) of 3.0) in 7H9 liquid media supplemented with AD. The cells were collected and suspended in PBS containing Tween 80 (0.05% (*v/v*) at an OD_600_ of 0.1. Further, bacteria were treated with 5- or 10-mM CHP (cumene hydroperoxide), 10-mM H_2_O_2_ (hydrogen peroxide), 200-ng MMC (mitomicin C), and 0.4% MMS (methylmethansulfate) and incubated for various amounts of time at 37 °C. Untreated cells suspended at an OD_600_ of 0.1 were used to control the viability of each strain and to determine the sensitivity of mutants to UV at doses of 5 mJ, 10 mJ, and 20 mJ. The colony-forming unit (CFU) methodology was applied to monitor the survival of strains treated with MMS or CHP. At the indicated time points, 100 μL of each cell suspension (tenfold diluted) was spread onto 7H10 solid agar supplemented with AD, and the plates were incubated for 72 h at 37 °C. In parallel experiments, serial dilutions of cell cultures treated with the indicated concentrations of MMS, H_2_O_2_, MMC, or CHP were spotted (5 µL) on 7H10 agar plates containing AD and incubated for 72 h at 37 °C. Each experiment was performed at least in triplicate.

### 2.5. Mutation Rate

To determine the mutation rate in *M. smegmatis* strains, an integrative pMV306Km plasmid containing the *sacB* gene was constructed (pMV306Km-sacB) and introduced by electroporation into competent cells of *M. smegmatis* mutants Δ*ku*, Δl*igD*, Δ*ku-ligD*, Δ*ligC1-ligC2-primC*, and Δ*ku-ligD-ligC1-ligC2-primC*. All mutants carrying pMV306Km-sacB were cultured in 7H9 medium containing 10% AD and kanamycin (25 µg) for 120 h (late-stationary phase) at 37 °C. Next, the cells were collected and suspended in PBS containing Tween 80 (0.05% (*v/v*) at an OD_600_ of 0.1. Each 100 μL of serial dilution (tenfold) was spread on two parallel plates, 7H10/AD medium containing kanamycin and 7H10/AD medium containing kanamycin and sucrose (2%). The mutation rate was calculated as the ratio of CFU on sucrose and kanamycin medium to CFU on kanamycin medium.

### 2.6. Next-Generation Sequencing 

Wild-type *M. smegmatis* and its mutants, as well as strains exposed to CHP, were analyzed by whole-genome sequencing (Table S3). The sequencing data for all strains are available as bioproject at the NCBI (National Center for Biotechnology Information) database under accession number PRJNA675073 and are shown in Table S4. The sequencing libraries were prepared using the Vazyme TruePrep DNA Library Prep Kit V2 for the Illumina preparation protocol (Vazyme Biotech Co., Ltd., Nanjing, China). A total of 1 ng of genomic DNA isolated from the wild-type strain and its 5 mutants, as well as 26 individual mutants exposed to CHP and 3 *M. smegmatis* cultures exposed to CHP (mixes), was used for the preparation of paired-end libraries, according to the manufacturer’s instructions. Whole-genome shotgun sequencing was performed on a NextSeq 500 platform at a read length of 2 × 150 bp (300 cycles). Raw sequencing reads were subjected to a quality check with FastQC v.0.11.8 [26]. Adapters and low-quality sequences were trimmed using trim_galore v.0.6.4 (http://www.bioinformatics.babraham.ac.uk/projects/trim_galore/, accessed on 8 April 2021). For wild-type and individual *M. smegmatis* mutants mapping reads, variant calling and variant annotation were performed by breseq v.0.35.4 [27] with default parameters. As a reference genome, *M. smegmatis* MC^2^ (NC_008596) was used. Common variants from wild-type and derived mutants were excluded from the final variant calling. Variant calling from *M. smegmatis* mutant mixes was performed with Snippy v.4.4.5 [28]. Variants with depths <10 and less frequent than 10% were excluded from further analysis. Common variants from the mutant mix and the corresponding wild type were excluded from the final variant calling. 

## 3. Results

### 3.1. NHEJ Proteins Promote the Resistance of Mycobacteria to DNA Methylation and Oxidation Assaults

**Figure 1:**
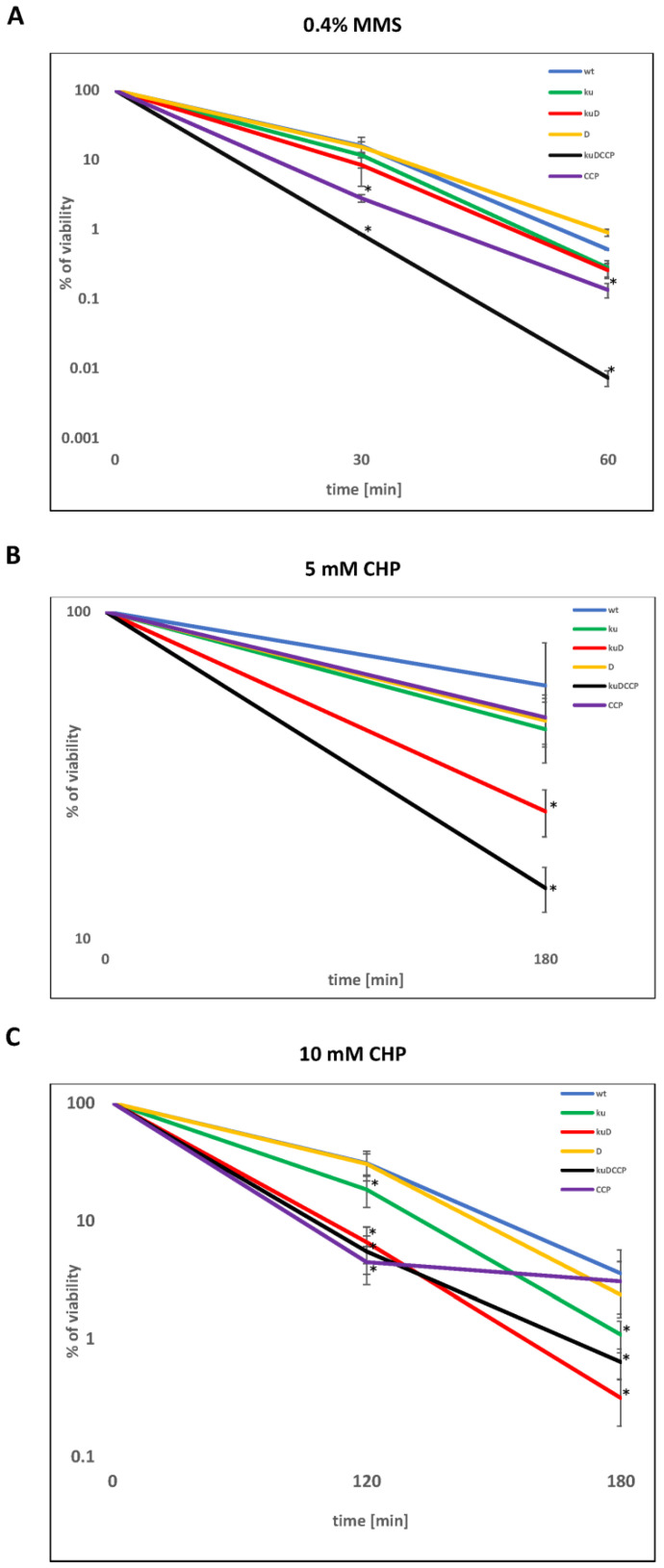
The viability of mycobacteria treated with oxidative and nitrosative agents. The survival of wild-type *Mycobacterium* (*Mycolicibacterium*) *smegmatis* (wt) and its mutants (ku-Δ*ku*, kuD-Δ*ku-ligD,* D-Δ*ligD*, kuDCCP-Δ*ku-ligD-ligC_1_-ligC_2_-primC*, and CCP-Δ*ligC_1_-ligC_2_-primC*) treated with methylmethansulfate (MMS) (**A**) or CHP (**B**) 5 mN or (**C**) 10 mM based on the colony-forming unit (CFU) analysis. The bacterial cells were treated with 0.4% MMS for 30 and 60 min (**A**), 5 mM of CHP for 180 min (**B**), and 10 mM of CHP for 120 min and 180 min (**C**). The percentage of survival was calculated by comparing the number of viable cells in treated vs. untreated samples from at least three independent experiments and plotted as the average +/- standard deviation. A Mann–Whitney rank-sum test was employed for comparisons of mutants versus the control samples (mc) to determine any significant differences between the mean values of the wild-type and mutant strains. The results were considered to be statistically significant (*) at *p* < 0.05.

Ionizing radiation and desiccation cause single- and double-stranded breaks in the DNA, which must be repaired by HR or NHEJ. It was already reported that NHEJ-deficient strains (Δ*ku*, Δ*ligD*, and Δ*ku*-*ligD*) of *M. smegmatis* are sensitive to IR during the stationary phase and that NHEJ protects mycobacteria against the harmful effects of desiccation [13]. Here, we evaluated the sensitivity of various NHEJ mutants to DNA-damaging assault-causing oxidative (cumene hydroperoxide, CHP, and hydrogen peroxide, H_2_O_2_) and nitrosative (methylmethanesulfonate, MMS) stress. Additionally, UV radiation and mitomycin C (MMC), known to induce an SOS response, were used as controls. The set of *M. smegmatis* mutants defective in the synthesis of the Ku protein, ATP-dependent ligase(s), and AEP-primase(s) comprising Δ*ku* (ku)*,* Δ*ku-ligD* (kuD), Δ*ligD* (D), Δ*ligC_1_-ligC_2_-primC* (CCP), and Δ*ku-ligD-ligC_1_ -ligC_2_-primC* (kuDCCP) were included in this experiment. The wild-type strain and the investigated mutants were cultured in a rich medium until the late-stationary phase (five days, OD_600_ 3.0) and then exposed to the several doses of DNA-damaging agents and plated on 7H10/AD at various dilutions. The double NHEJ mutant Δ*ku-ligD* and the mutant defective in the synthesis of all investigated proteins (kuDCCP) were most sensitive to all tested DNA-damaging assaults, including CHP, H_2_O_2_, UV, and MMS (Figure S2). The growth of the CCP mutant was affected by CHP compared to the wild-type strain. The viability of all investigated strains was monitored more precisely after their exposure to MMS and CHP by using a colony-forming unit (CFU) analysis. After 30 and 60 min of treatment with MMS, the number of viable cells decreased significantly more (*p* = 0.006 and *p* < 0.001, respectively) in the mutant defective in the synthesis of all investigated proteins (*Δku-ligD-ligC_1_-ligC_2_-primC*) compared to the wild-type strain (Figure 1A). However, the number of viable cells of the considered mutants (*Δku, Δku-ligD*, and *ΔligC_1_-ligC_2_-primC*), after 60 min of treatment with MMS, was not statistically different (*p* = 0.505, 0.541, and 0.094, respectively) compared to the wild-type strain. On the other hand, the viability of the *ΔligC_1_-ligC_2_-primC* mutant decreased significantly after 30 min of treatment with the MMS compared to the wild-type strain (*p* < 0.001). The double-mutant KuD and KuDCCP strains were sensitized significantly after treatment with 5-mM CHP (*p* = 0.019 and *p* < 0.001, respectively, Figure 1B) and 10-mM CHP for 120 min (*p* = 0.001 and *p* < 0.001, respectively, Figure 1C) or prolonged treatment with CHP for 10 mM/180 min (*p* = 0.019 and *p* < 0.001, respectively, Figure 1C). The strain defective in the synthesis of the Ku protein was sensitized to CHP at a concentration of 10 mM, both at 120 and 180 min (*p* = 0.008, *p* = 0.013, respectively, Figure 1C). Mutant *ΔligC_1_-ligC_2_-primC* was significantly sensitized only after treatment with 10-mM CHP at 120 min. (*p* = 0.001, Figure 1C). 

### 3.2. The Presence of LigD and LigC1/C2/PrimC Increases the Frequency of Mutations in Mycobacteria

**Figure 2:**
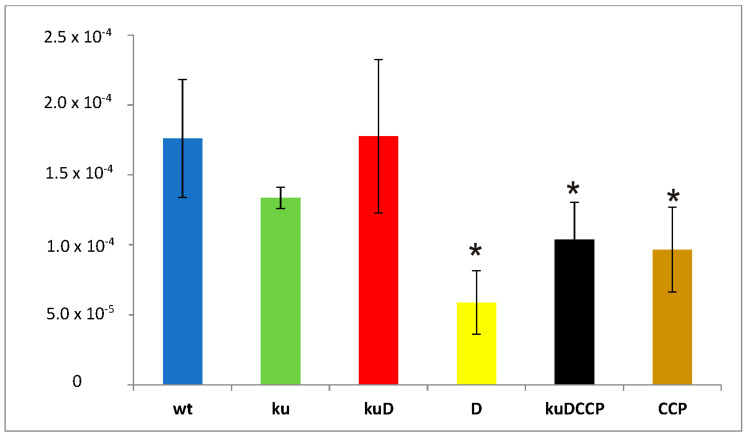
Number of sucrose-resistant mutants identified in wild-type *M. smegmatis* (wt) and all analyzed mutants (ku-*Δku,* kuD-*Δku-ligD,* D-*ΔligD,* kuDCCP-*Δku-ligD-ligC_1_-ligC_2_-primC,* and CCP-*ΔligC_1_-ligC_2_-primC*). The number of viable cells was calculated based on CFU enumerations from three independent experiments and plotted as the average ± standard deviation. One-way analysis of variance (ANOVA) (Holm–Sidak method) was employed for multiple comparisons versus the control samples (mc) to determine any significant differences between the mean values of the wild-type and mutant strains. The results were considered to be statistically significant (*) at *p* < 0.05.

The repair of DSBs by the NHEJ pathway might be mutagenic if primases are involved in the processing of the broken DNA ends. It has not yet been determined whether the involvement of ATP-dependent ligases and AEP primases in base excision repair is a faithful process. We evaluated the frequency of spontaneous mutations appearing in wild-type *M. smegmatis* and mutants growing in the stationary phase. To monitor the appearance of mutations, all the strains were enriched with the reporter *sacB* gene introduced with the integration plasmid into the *attB* site of chromosomal DNA. The presence of *sacB* sensitizes the host strain to sucrose. The number of spontaneous sucrose-resistant mutants of all strains carrying the inactivated *sacB* gene was counted on 7H10/AD plates supplemented with sucrose and compared to the number of colonies counted on control, sucrose-free plates. The frequency of mutations responsible for the acquired resistance of the tested strains to sucrose was significantly lower in Δ*ligD*, Δ*ligC_1_-ligC_2_-primC*, and Δ*ku-ligD-ligC_1_-ligC_2_-primC,* with *p* = 0.002, *p* = 0.004, and *p* = 0.004, respectively (Figure 2). 

Next, 35/36 mutants of each sucrose-resistant strain were analyzed to identify the molecular mechanism of acquired resistance. From 60% to 80% of sucrose-resistant mutants presented a partial deletion of the *sacB* gene, as identified by PCR and Southern hybridization (Figure S3). The remaining colonies accumulated insertion or the deletion of a single nucleotide A (all strains) or a few nucleotides (insertion of AATGA in Δ*ligD* and deletion of GAC in Δku-*ligD*). The inversion of 670 bp of *sacB* was determined in Δ*ligC1-ligC2-PrimC*. Nucleotide substitutions were detected in all strains except Δ*ku*. The substitutions T/C and C/T were identified in the wild-type strain and Δ*ku*-*ligD*. The substitutions identified in the remaining strains (CCP and kuDCCP) were A/G and T/G. 

### 3.3. LigD/Ku Promotes Double Substitutions and Single Nucleotide Insertions in the Presence of a DNA-Oxidizing Agent

Oxygen radicals may attack DNA at either the sugar or the base, leading to sugar fragmentation, base modification or loss, and strand breaks. Alkyl hydroperoxides, such as cumene hydroperoxide, are of moderate-to-high concerns in terms of potential carcinogenicity, because they are more stable than other peroxides. Here, we used the CHP treatment of mycobacterial cells to analyze the role of ATP-dependent DNA ligases and AEP primases in repairing the DNA damage caused by oxygen radicals. The wild-type *M. smegmatis* strain, as well as the mutants Δ*ligC_1_-ligC_2_-primC* and *Δku-ligD-ligC_1_-ligC_2_-primC*, were cultured in rich media (7H9/AD) supplemented with 0.5-mM CHP until the late-stationary phase and plated on 7H10/AD plates. Then, six randomly selected colonies of the wild-type strain and ten randomly selected colonies of each mutant were subjected to DNA isolation and sequencing using next-generation sequencing (NGS) technology. The DNA of the initial wild-type strain and both mutants, cultured without supplementation of CHP, were also used for the NGS analysis as a control. The sequence of each genome was mapped to the genome of *M. smegmatis* mc^2^ (NC_008596) to identify the substitutions, insertions, and deletions of nucleotides. Common variants identified in the control strains, as well as strains treated with CHP, were excluded from the final variant calling. Finally, the pattern of the unique nucleotide substitutions, deletions, and insertions identified in six wild-type colonies and 10 colonies of each mutant strain growing in the presence of CHP are summarized in Table 1 (see details in Supplementary File, Table S4). The single-nucleotide substitution pattern was not dramatically changed in the strains defective in the synthesis of NHEJ proteins. On the other hand, the double-nucleotide substitution TC→CT appeared efficiently (50% genomes of the wild type and 40% genomes of CCP vs. 0% of KuDCCP) in strains carrying the functional Ku and LigD proteins, exclusively. It is also worth noting that CA→AC substitutions were exclusively identified in the wild-type strains (33.3%). Similarly, the CG insertion identified in approximately 30% of the strains carrying the functional *ligD* and *ku* genes was not determined in the KuDCCP mutant. The most common deletion of a single nucleotide was missing G and this ΔG mutation was especially abundant (40% of genomes) in the KuDCCP mutant.

**Figure 3:**
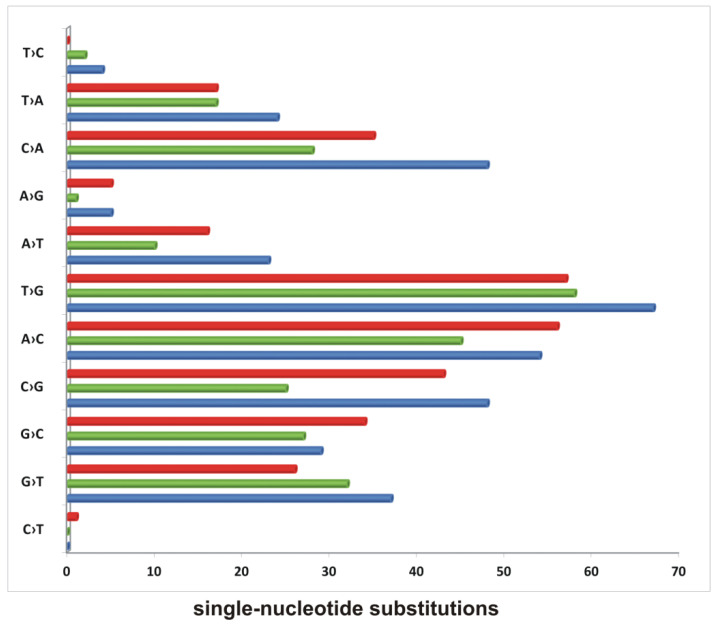
The accumulation of single-nucleotide substitutions in mycobacterial strains growing in the presence of cumene hydroperoxide. The number of SNPs identified in wild-type *M. smegmatis* (blue), *Δku-ligD-ligC_1_-ligC_2_-primC* (red), and *ΔligC_1_-ligC_2_-primC* (green) growing in the presence of CHP identified by sequencing the mixture of cells.

To quantitate the single-nucleotide substitutions in the wild-type strain versus mutants, cultures of investigated strains growing in the presence of CHP were directly subjected to DNA isolation and sequencing. Since we expected a mixture of different mutants in each DNA sample, the sequencing was deep with high coverage (20 million reads per sample). Variants with depths <10 and less frequent than 10% were not included in our analysis. In this method, we were not able to reliably determine insertions and deletions. The data analysis revealed 339 single-nucleotide substitutions in the DNA isolated from the culture of the wild-type strain, 245 single-nucleotide substitutions in the *ΔligC_1_- ligC_2_-primC* mutant*,* and 290 single-nucleotide substitutions in the *Δku-ligD-ligC_1_- ligC_2_-primC* mutant (Figure 3 and Table S5). 

The most abundant substitutions in all strains were T→G and A→C. The substitutions C→G and C→A were abundant in the wild-type strain and KuDCCP and decreased by approximately 50% in the CCP mutant. The mutant CCP also cumulated less than 50% of the A→T substitutions determined in the wild-type strain. Two substitutions, G→C and A→C, were overrepresented in the KuDCCP mutant compared to in the wild-type strain.

## 4. Discussion

The role of NHEJ in the DSB repair process in mycobacteria is well-described. NHEJ is a mutagenic process if the DNA ends need processing before sealing, resulting in the insertion or deletion of a few nucleotides. End processing is performed by LigD displaying phosphodiesterase, polymerase, and ATP-dependent ligase activity [29,30]. The DNA end-sealing activity of LigD could also be replaced with an alternative ATP-dependent ligase, LigC [18]. More recently, it was reported that PrimC composes a complex with BER proteins and fills short 1-3 nucleotide gapped DNA intermediates with ribonucleotides, and LigC ligates the resulting nicks to complete repairs. The copurification of Prim-PolC and LigD, but not Ku, on a substrate containing a single nucleotide gap, was also reported. Mutants defective in the synthesis of LigC-PrimC or LigD appeared to be sensitive to oxidative DNA damage [24]. More recently, the crystal structures of Prim-PolC, bound to gapped DNA substrates, demonstrated the Prim-PolC-binding preference for short gaps and revealed their operating mechanism, which ensures that the unpaired templating bases in the gap are settled into the active site in an ordered manner [31].

Both LigD and PrimC can extend templated and nontemplated ribonucleoside triphosphates (rNTPs) to DNA substrates [14,15]. This might suggest that the BER pathway engaging LigC-PrimC or LigD to fill in the gaps is mutagenic; however, it remains to be further elucidated. 

The BER process is initiated by the detection and removal of nonbulky and nonhelical distorting lesions by a specific N-glycosylase [3]. Subsequently, the AP site is processed by AP lyases or AP endonucleases and, further, by exonucleases at the 3′-end and dRPases at the 5′-dRP terminus to leave ligatable 3’-OH and 5′-P termini [3,32]. It was shown that human Ku is essential for the efficient removal of AP sites localized in the proximity of DSBs and presents 5′dRP/AP lyase activity, resulting in nicking DNA at the 3′-end of an abasic site [33]. Further, the AP/5′-dRP lyase activity was shown for the Ku protein of *Bacillus subtilis* supporting the efficient joining of DNA ends with abasic sites at their termini [34]. It was also reported that LigD of *B. subtilis* presents 5′-dRP lyase activity at its N-terminal ligase domain. The AEP primase and ATP ligase, together with the 5′-dRP lyase activity of *B. subtilis* LigD, allow the efficient in vitro repair of DNA containing 2′-deoxyuridine [35]. 

In the stationary phase, under growth-restricting conditions, the bacterial population rapidly evolves accumulating mutations that may allow the whole population to adapt to the environment [36]. NHEJ enzymes were reported in mycobacteria as contributing to DSBs repair during a prolonged stationary phase, while under desiccation conditions [13], in tubercle bacilli deposited in human macrophages [37] and in *Bacillus subtilis* during the sporulation process [38]. NHEJ enzymes were also evaluated in regard to the frequency and spectrum of mutations in starving *Pseudomonas putida* [39]. The authors applied a plasmid reporter system, allowing the identification of various base substitutions and in-frame deletion eliminating the stop codon from *pheA* gene [40] to monitor the frequency and spectrum of mutations in various NHEJ-deficient strains. The frequency of mutations was not significantly affected in *ku, ligD,* and *ku-ligD* mutants, nor in mutants defective in the POL or PE domains. On the other hand, the spectra of stationary phase mutations differed between the wild type and mutants, as well as, between the various mutants. The authors concluded that LigD and Ku participate in a mutagenic process in starving *P. putida* and that LigD acts in the stationary-phase mutagenesis independently from Ku [39]. 

We generated a set of *M. smegmatis* mutants defective in the synthesis of core or alternative NHEJ proteins and exposed them to various DNA-damaging assaults. Strains defective in the synthesis of Ku, including single Δ*ku,* double-mutant, Δ*ku-ligD*, and mutant defective in the synthesis of all investigated proteins, Δ*ku-ligD-ligC_1_-ligC_2_-primC,* appeared to be more sensitive to MMS, known to methylate DNA, predominantly on N7-deoxyguanosine and N3-deoxyadenosine, causing base mispairing, which is preferentially repaired by BER [41]. The CFU analysis indicated the Δ*ku-ligD-ligC_1_-ligC_2_-primC* mutant as the most sensitive to MMS; however, the prolonged exposition to MMS also revealed the sensitization of Δ*ligC_1_-ligC_2_-primC* compared to the wild-type strain. The far lesser sensitization to MMS observed for Δ*ku-ligD* and Δ*ligD* might suggest that the effect does not arise from the inhibition of NHEJ in the analyzed mutants but, rather, a NHEJ-independent function of the investigated proteins. Single-strand lesions and single-strand breaks that are generated in the process of BER are also potential sources of DSBs if they appear in close proximity. HR-deficient strains are sensitive to MMS, so it was postulated that a treatment with MMS leads to DSB accumulation [42,43]; however, it is not clear if the sensitivity is due to DSB accumulation or replication fork stoppage [44]. It was also reported that the accumulation of lesions caused by alkylation damage (3-methylcytosine) is preferentially localized at ssDNA and that the unrepaired damage accumulated in ssDNA induces hypermutability at DSBs [45]. The sensitivity of the mutants analyzed here to MMS suggests the engagement of both the Ku protein and AEP-dependent primases in the repair of alkylation damage in mycobacteria. The fact that mutants defective in the synthesis of all investigated proteins were more susceptible to MMS than Δ*ku* and Δ*ku-ligD* suggests that DSBs are not the only lesions repaired by ATP ligases and AEP primases under treatment with mycobacteria by MMS. A wide range of oxidized nucleobases are caused by hydroxyl radicals. The particularly susceptible base to oxidative DNA damage is guanine, which results in the accumulation of 8-oxo-7,8-dihydroguanine (8oxoG). Hydroxy radicals might also produce 5-hydroxycytosine, 4,6-diamino-5-formamidopyrimidine (FapyA), and 2,6-diamino-4-hydroxy-5-formamidopyrimidine (FapyG) [3]. The creation of an abasic site and, consequently, single- or double-stranded breaks might also appear during oxidative stress. We applied H_2_O_2_ and CHP to evaluate the potential role of NHEJ proteins in repairing oxidative damage in mycobacteria. All mutants defective in the synthesis of the Ku protein were the most sensitive to oxidative stress. Furthermore, exposure to a higher concentration of CHP was also toxic to *ΔligC1*-*ligC2*-*primC* compared to the wild-type strain. On the other hand, the single mutant Δ*ligD* was not much affected by oxidative agents compared to the control strain. DSBs generated by oxidative damage could explain the phenotype of Δ*ku* or Δ*ku*-*ligD*; however, it should also affect the single Δ*ligD* mutant. Further, Δ*ligC1-ligC2-primC,* which is sensitized in the presence of CHP, is not defective in the repair of DSBs. Taking this into consideration, we postulate that Ku, ATP-dependent ligases, and AEP primases are BER-associated proteins involved in the repair process of oxidized bases, nicks, or abasic sites likely in proximity to DSBs. 

The NHEJ pathway repairing DSBs is mutagenic if the ends require processing before sealing. The potential mutability of NHEJ-related proteins in BER has not yet been investigated. To explore the significance of NHEJ-related proteins in the accumulation of spontaneous mutations, we assessed the frequency of mutations appearing in the *sacB* gene (sucrose sensitivity) of the wild-type strain and mutants growing in the stationary phase. In agreement with Paris and colleagues [39], we found that inactivation of *ku* or *ku* and *ligD* did not affect the frequency of the mutations in the conditions of the experiment. On the other hand, the inactivation of *ligD* alone, *LigC_1_LigC_2_PrimC,* or all investigated proteins together decreased the number of selected sucrose-resistant mutants. The majority of sucrose-resistant mutants (60–80%) were obtained by deleting the *sacB* gene or its part, which might not be related to the proteins investigated. The remaining mutants accumulated insertions, deletions, and substitutions of a single nucleotide in *sacB,* but we were not able to relate the spectra of mutations to a specific genotype. 

Further, we analyzed the mutations appearing under the pressure of oxidative stress (CHP treatment) in the wild-type strain and mutants (Δ*ku-ligD-ligC_1_-ligC_2_-primC* and Δ*ligC_1_-ligC_2_-primC*) growing in the stationary phase. The genomes of the individual colonies of the wild-type strain and mutants were isolated and sequenced. The double substitutions of two nucleotides (CA/AC and TC/CT) depended on the presence of a functional LigD and Ku and were not identified or identified at a much lower frequency in Δ*ku-ligD-ligC_1_-ligC_2_-primC* compared to the wild-type strain and Δ*ligC_1_-ligC_2_-primC* mutant. Two-nucleotide insertions (CG) were also identified with high frequency in the wild-type strain and Δ*ligC_1_-ligC_2_-primC* mutant but not in the strain defective in the synthesis of Ku/LigD proteins. It is likely that CG insertion as well as CA/AC and TC/CT substitutions are related directly to the unfaithful NHEJ repair process. On the other hand, the frequency of the G deletion increased significantly (40% of genomes vs. 10% of genomes) in the mutant defective in the synthesis of Ku/LigD, suggesting that the presence of core NHEJ proteins protects G nucleotides (or 8oxoG) from deletion. The number of single-nucleotide substitutions in the individual genomes was too low to quantitate a difference between strains. However, it might be interesting that G→C, G→T and C→T were substitutions that appeared in some mutant genomes but not in the wild-type strain. One can speculate that such substitutions, especially G→T and C→T, are linked to 8oxoG modification, protected somehow by the presence of NHEJ-related proteins. The quantitative analysis of the single-nucleotide substitutions identified in chromosomal DNA isolated directly from the cultures of strains growing in the presence of CHP showed a decrease in the number of substitutions in mutant strains compared to the control. The substitutions whose numbers decreased most significantly in the Δ*ligC_1_-ligC_2_-primC* mutant were C→A, A→T and C→G. On the other hand, the substitution G→C was more abundant in both mutants and G→T in the Δ*ligC_1_-ligC_2_-primC* strain, confirming the observation made with the individual mutants and suggesting the role of LigC_1,2_PrimC in the processing of 8oxoG. 

AEP primases and ATP-dependent ligases are involved in the repair process of DSBs, and, possibly, other lesions of DNA during the stationary phase, starvation, and desiccation in the limited access to carbon sources and oxygen when bacteria are in a nonreplicating stage. The enzymes utilize ribonucleotides, allowing the repair of damage when the dNTP pool is limited. The DNA repair process carried out by NHEJ-related enzymes is unfaithful and leads to the accumulation of mutations. The life cycle of tubercle bacilli includes the stage inside phagosomes, exposition to reactive nitrogen and oxygen species, prolonged states of persistence, and limiting access to oxygen and nutrients in granulomas. Such conditions promote the use of AEP primases and ATP-dependent ligases in DNA repair with the expected accumulation of mutations. The mutations accumulated in the bacterial population might contribute to the acquisition of resistance to anti-TB drugs used in the therapy of tuberculosis. The resistance of the clinical *Mtb* to first- and second-line anti-TB drugs is based mainly on the accumulation of point mutations in genes encoding drug-targeted proteins or enzymes activating the prodrug inside bacilli (for a recent review, see [46]). However, the real contribution of NHEJ-related proteins to the development of acquired resistance to anti-TB drugs in clinical *M. tuberculosis* strains remains to be elucidated.

## 5. Conclusions

All data collected here and already published by other authors lead to the conclusions that NHEJ-related proteins control the damage caused by toxic radicals but by unfaithful DNA repair mechanisms. The presence of NHEJ-related proteins increases the mutation frequency in the mycobacterial genome but protects the viability of cells. NHEJ-related proteins are involved in the repair process of oxidized/methylated bases, at least in proximity to DSBs. 

## Figures and Tables

**Table 1:** Mutations detected in the individual colonies of *Mycobacterium* (*Mycolicibacterium*) *smegmatis* cells growing in the presence of cumene hydroperoxide (CHP).

	*Mycobacterium Smegmatis Strains*		
	Wild-Type	KuDCCP	CCP
Substitution	% of Genomes Affected		
A→C	16.67%	-	10.00%
A→G	16.67%	-	-
A→T	-	-	-
C→A	16.67%	10.00%	-
C→G	16.67%	10.00%	10.00%
C→T	-	20.00%	10.00%
G→A	33.33%	10.00%	20.00%
G→C	-	10.00%	20.00%
G→T	-	10.00%	10.00%
T→A	-	10.00%	-
T→C	16.67%	-	10.00%
T→G	33.33%	10.00%	10.00%
CA→AC	33.33%	-	-
TC→CT	50.00%	-	40.00%
TA→CT	-	-	10.00%
CAAC→GGTG	16.67%	-	-
**deletion**	**% of genomes affected**		
ΔA	16.67%	-	-
ΔC	-	-	-
ΔG	16.67%	40.00%	10.00%
ΔT	-	-	-
**insertion**	**% of genomes affected**		
T	16.67%	-	-
CG	33.33%	-	30.00%

Six colonies of wild-type *M. smegmatis* and 10 colonies of each mutant Δku-ligD-ligC1-ligC2-primC (KuDCCP) and ΔligC1-ligC2-primC (CCP) growing in the presence of CHP were sequenced using next-generation sequencing (NGS) technology and analyzed for the presence of mutations. The most abundant changes are highlighted by a gray background.

## Data Availability

The data presented in this study are available on line (see supplementary files and NCBI data base) or on request from the corresponding author.

## Supplementary Materials

The following are available online at https://www.mdpi.com/article/10.3390/genes12040547/s1, Figure S1: Southern blot analysis of the Δku-ligD-ligC1-ligC2-prim mutant, Figure S2: Phenotypic analysis of M. smegmatis strains in the presence of various DNA damaging assaults, Figure S3: Southern blot analysis of sucrose-resistant mutants, Table S1: Bacterial strains, Table S2: Plasmids and primers, Table S3: Description of strains sequenced by NGS (separate excel file), Table S4: Summary of NGS analysis (separate excel file), Table S5: Mutations detected in *M. smegmatis* cells growing in the presence of CHP.
