# Supplementary material for: ATP-Dependent Ligases and AEP Primases Affect the Profile and Frequency of Mutations in Mycobacteria under Oxidative Stress
Source: Genes (Basel). 2021 Apr 9;12(4):547. doi: 10.3390/genes12040547 (PMC8068969; doi:10.3390/genes12040547)
Supplement: Supplementary file 1 [file genes-12-00547-s001.zip › genes-1139527-SI.pdf]

ATP-dependent ligases and AEP primases affect the profile and frequency of mutations in mycobacteria under oxidative stress.

Anna Brzostek, Filip Gąsior, Jakub Lach, Lidia Żukowska, Ewelina Lechowicz, Małgorzata Korycka-Machała, Dominik Strapagiel, Jarosław Dziadek

Supplementary Figures and Tables:

Tables:

1. Table S1 – Bacterial strains
2. Table S2 – Plasmids and primers
3. Table S3 –Description of strains sequenced by NGS (see separate data set, Excel)
4. Table S4 - Summary of NGS analysis (see separate data set, Excel)
5. Table S5 – Mutations detected in *M. smegmatis* cells growing in the presence of CHP

Figures:

1. Figure S1 – Southern blot analysis of the Δ*ku-ligD-ligC1-ligC2-prim* mutant
2. Figure S2 – Phenotypic analysis of *M. smegmatis* strains in the presence of various DNA damaging assaults
3. Figure S3 – Southern blot analysis of sucrose-resistant mutants

Table S1 – Bacterial strains

Name/genotype	STRAINS	Reference
Top10F'	*Escherichia coli* strain	Invitrogen
mc^2155	*M. smegmatis* - wild type	Laboratory stock
Δku	*M. smegmatis* defective in the synthesis of Ku	Korycka-Machala et al., 2007; Pitcher et al., 2007
ΔligD	*M. smegmatis* defective in the synthesis of LigD	Korycka-Machala et al., 2007; Pitcher et al., 2007
Δku-ligD	*M. smegmatis* defective in the synthesis of Ku and LigD	Korycka-Machala et al., 2007; Pitcher et al., 2007
ΔligC₁-ligC₂-primC	*M. smegmatis* defective in the synthesis of $LigC_1$, $LigC_2$ and PrimC	Plocinski et al., 2017
Δ ku-ligD-ligC₁-ligC₂-primC	*M. smegmatis* defective in the synthesis of Ku, LigD, $LigC_1$, $LigC_2$ and PrimC	This study

Table S2 - Plasmids and primers

**Plasmids used for this study**	**CLONING VECTORS**	
pJET 1.2/blunt	Blunt cloning vector, AmpR	Thermo Scientific
p2NIL	Recombination vector, nonreplicating in mycobacteria, Kan^R	Parish& Stoker, 2000
pGoal17	Source of PacI cassette and *sacB,* Amp^R	Parish& Stoker, 2000
pMV306K	Mycobacterial integrative vector, Kan^R	Med-Immune Inc
pMV306Km-sacB	Integrative plasmid for selection of sucrose resistant ($sacB^R$) colonies	This study
**Primers used in this study**		
Name of primer	Sequence (5'>3')	Application
Msmeg_ligC1,ligC2, Prim2 probe-F	TCACTGCTTGCGGTTGCCCTGC	Southern blotting - hybridization probe
Msmeg_ligC1,ligC2, Prim2 - probe-R	GGCGAGCGGTTGTTCATCG	
sacBNotI-F	CGCGGCCGCTCGAGTGACCACAACGACGC	*sacB* cloning into pMV306Km
sacB XbaI-R	CTCTAGATCGGCATTTTCTTTTGCGTTT	

Table S3 – Description of strains sequenced by NGS (see separate data set, Excel)

Table S4 – Summary of NGS analysis (see separate data set, Excel)

Table S5 - Mutations detected in *M. smegmatis* cells growing in the presence of CHP.

	M. smegmatis strains		
	**wild-type**	**CCP**	**KuDCCP**
**substitution**	**number of positions affected**		
C→T	0	0	1
G→T	37	32	26
G→C	29	27	34
C→G	48	25	43
A→C	54	45	56
T→G	67	58	57
A→T	23	10	16
A→G	5	1	5
C→A	48	28	35
T→A	24	17	17
T→C	4	2	0
G→A	0	0	0
total	339	245	290

The culture of wild-type *M. smegmatis* as well as mutants Δ*ku-ligD-ligC1-ligC2-primC* (KuDCCP) and Δ*ligC1-ligC2-primC* (CCP) growing in the presence of CHP were sequenced using NGS technology and analyzed concerning the presence of mutations. The most abundant changes are highlighted by gray background.

Figure S1 - Southern blot analysis of the Δ*ku-ligD-ligC1-ligC2-prim* mutant

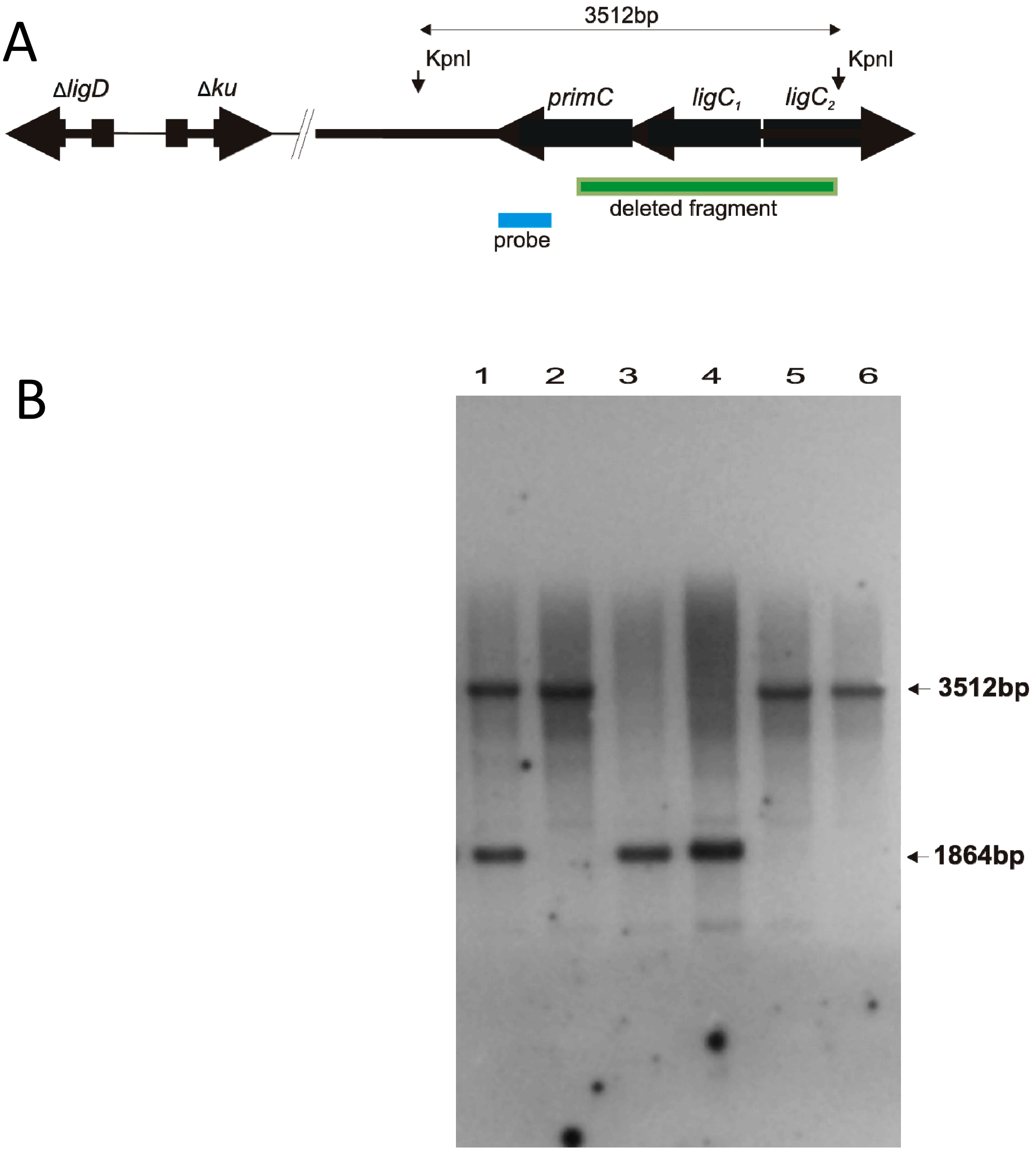

Fig. S1. Confirmation of mutant construction by Southern blotting analysis. (**A**) Genomic DNA fragment containing *ligC*$_1$ (1056 bp), ligC$_2$ (1065 bp) and *primC* (1116 bp) as well as mutated genes Δ*ku* and Δ*ligD* (genetic background of the strain for mutagenesis) are shown. KpnI restriction sites, internal deletion fragment and genetic probe are indicated. **(B)** Southern blot autoradiogram shows the presence of the intact genes (*ligC*$_1$-ligC$_2$-*primC*) in the wild type strain (**lane 2;** 3512 bp), both intact and mutated genes in the single crossover mutant (**lane 1;** 3512 and 1864 bp), mutated genes in the double crossover mutants (**lanes 3-4;** 1864 bp), and wild-type genes in the double crossover strains reversed to the wild-type genotype (**lanes 5-6;** 3512 bp).

Figure S2 – Phenotypic analysis of *M. smegmatis* strains in the presence of various DNA damaging assaults

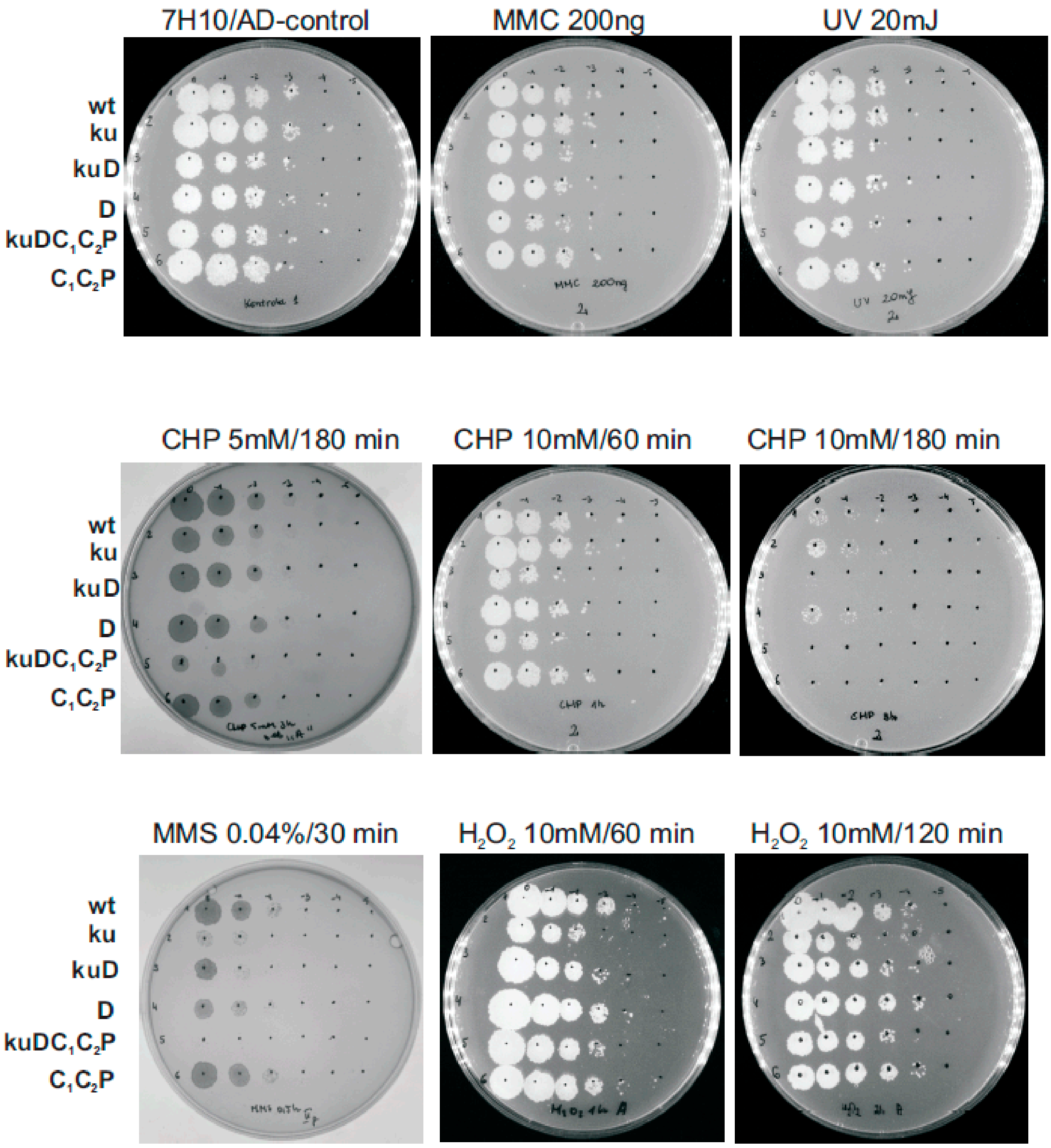

Fig. S2. The survival of *M. smegmatis* wild type (wt) and mutant cells: Δ*ku* (ku); Δ*ku-ligD* (kuD); Δ*ligD* (D); Δ*ku-ligD ligC1-ligC2-PolC* (kuDC1 C2P) and Δ*ligC1-ligC2-PolC* (C1C2P) in the absence of DNA damage (7H10/AD control) and after treatment with MMC, UV, CHP, MMS and H_2O_2. For qualitative analysis, the 5µl of indicated dilutions of cells (10^0-10^{-5}) were plated and after 3 days of incubation the plates were photographed using the gel documentation system.

Figure S3 – Southern blot analysis of sucrose resistant mutants

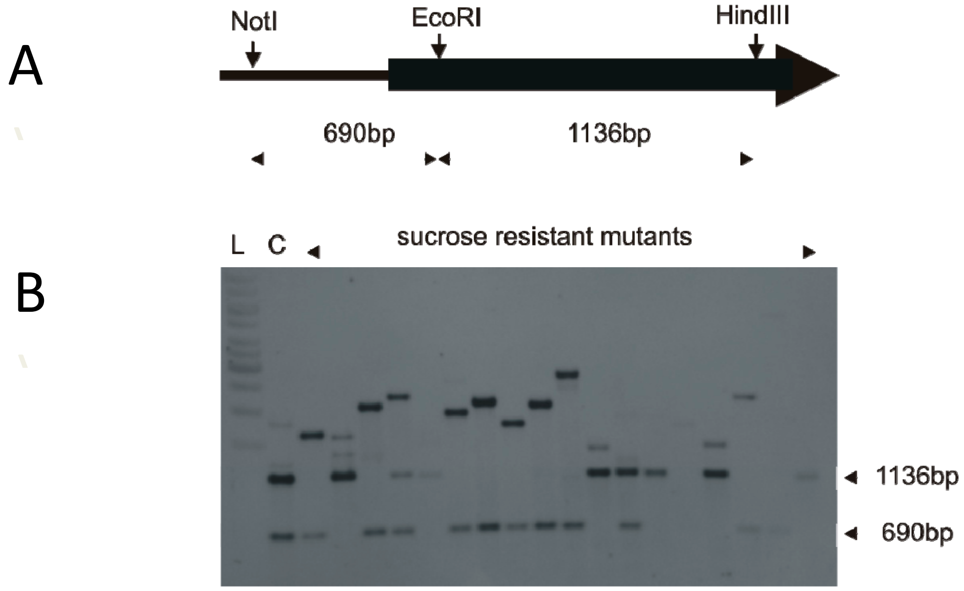

Fig. S3. The analysis of *sacB* gene by Southern blotting. (**A**) The black arrow represents *sacB* gene and its promoter introduced into the chromosomal DNA of *M. smegmatis* strain. The restriction sites and DNA fragments generated by digestion of chromosomal DNA by NotI/EcoRI/HindIII are indicated. **(B)** Southern blot autoradiogram shows the presence of the fragments of *sacB* gene and its promoter in the wild-type strain (C; 1136 and 690 bp) and selected sucrose-resistant mutants. L represents 1kb-DNA ladder.
