# Supplementary figures and images for: ATP-Dependent Ligases and AEP Primases Affect the Profile and Frequency of Mutations in Mycobacteria under Oxidative Stress
Source: Genes (Basel). 2021 Apr 9;12(4):547. doi: 10.3390/genes12040547 (PMC8068969; doi:10.3390/genes12040547)

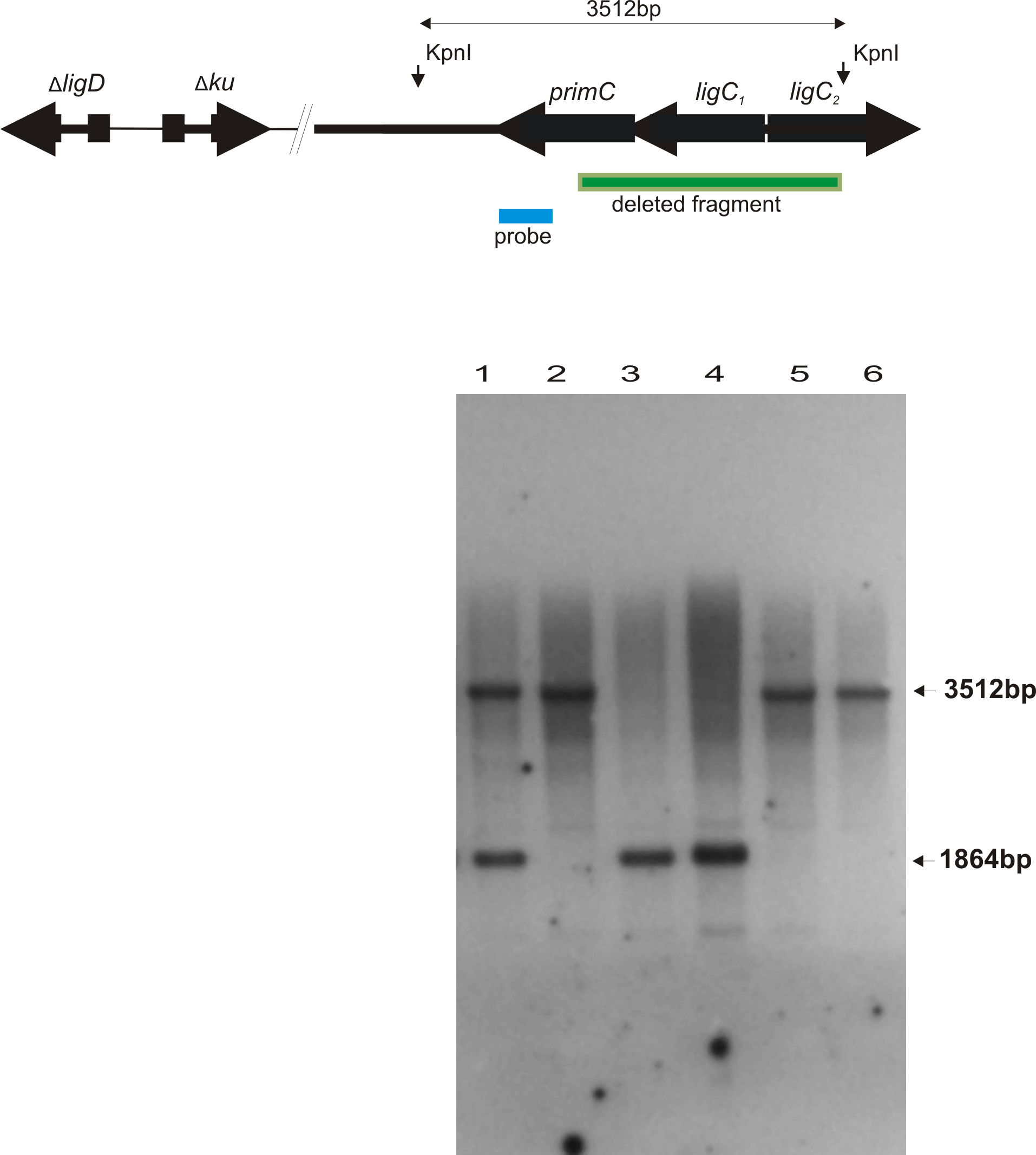

Supplement: Supplementary file 1 [file genes-12-00547-s001.zip › Figure S1-Southern.tif]

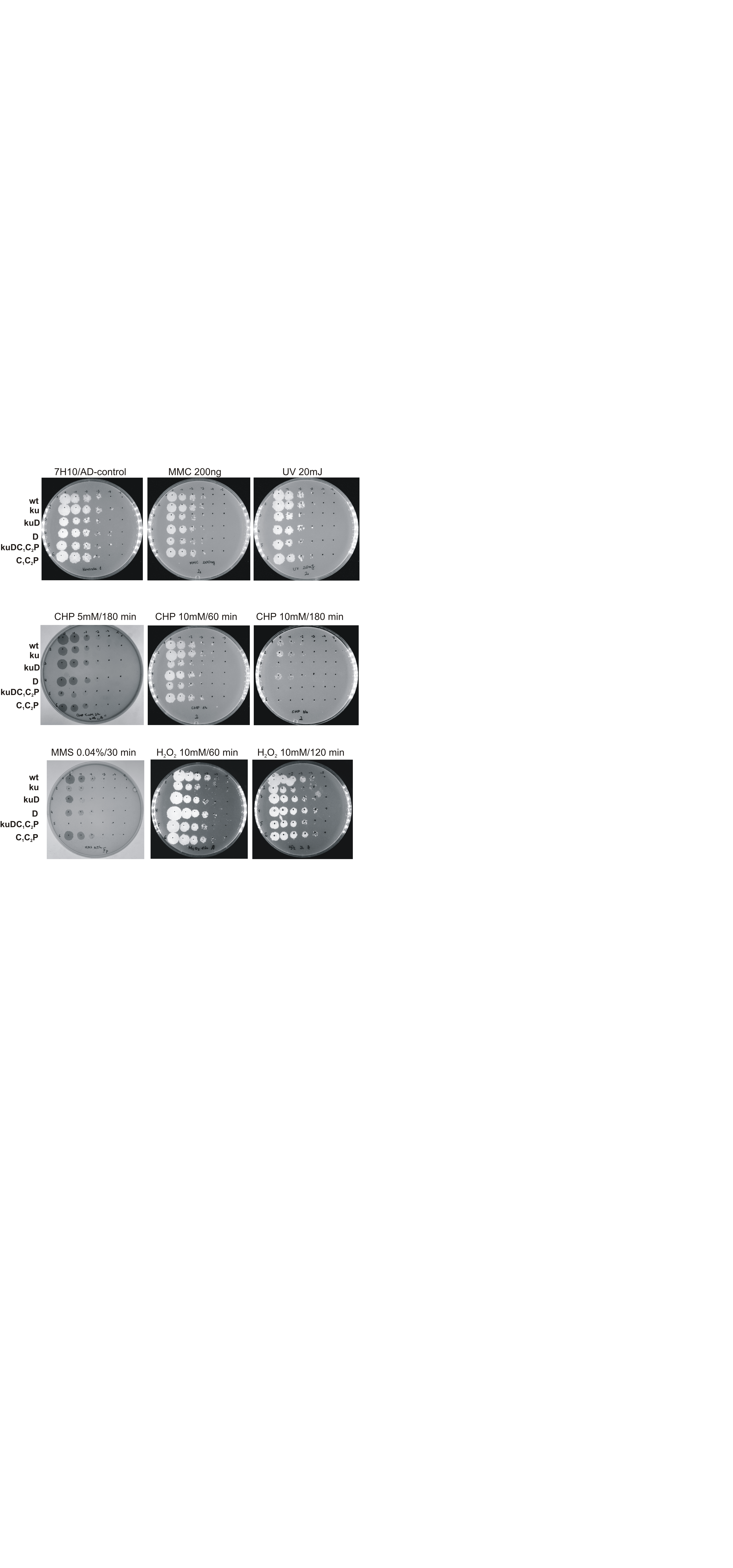

Supplement: Supplementary file 1 [file genes-12-00547-s001.zip › Figure S2-plates.tif]

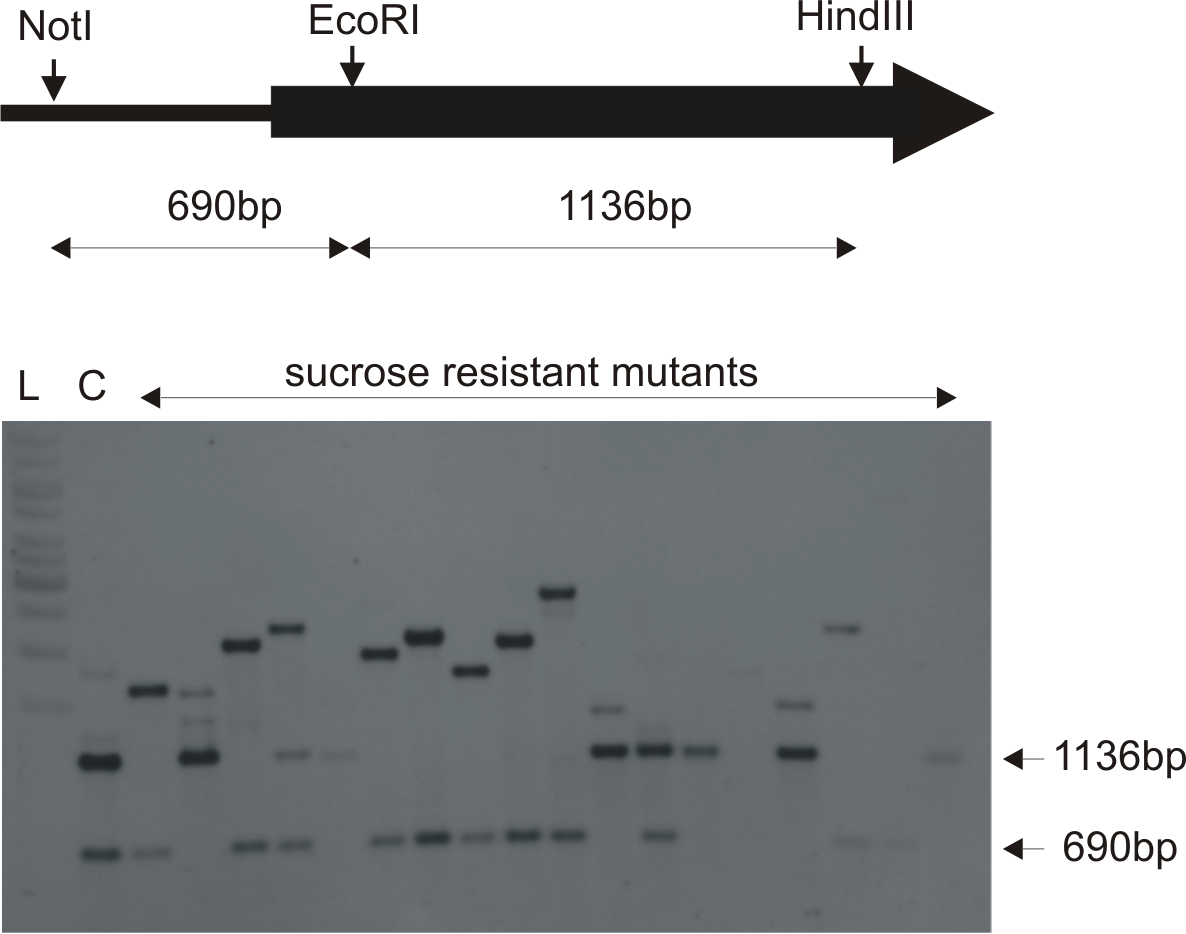

Supplement: Supplementary file 1 [file genes-12-00547-s001.zip › Figure S3-sucrose.TIF]
